# Surgery

**Published:** 1877-11

**Authors:** 


### II. Surgery.

A Fenestrated Bandage for the Leg, After Pirogoff's Amputation.—Prof. Dittel. (*Wiener Med. Wochenschrift*, 1877, No. 16.)—The piece of the os calcis, which after Pirogoff's amputation is to unite with the lower end of the tibia, has a great tendency to slide down, and it taxes the surgeon's attendance a good deal to prevent such displacement. Most surgeons think this displacement is caused by the tractions of the tendo Achillis. Dittel, however, is of the opinion that it is simply caused by the weight of the bone; as the patient usually is lying on his back, the os calcis is pulled downward by its own weight. D. thinks this displacement can be obviated by the following dressing: the bones being well adjusted and the edges of the wound being united by catgut sutures, the proper position of the os calcis is temporarily ecured by a broad strip of adhesive plaster, which, applied to the calf of the leg, is carried down and over the stump to the anterior aspect of the leg, to terminate at the crista tibiæ. The permanent bandage is then made with several strips of a plaster-of-Paris roller, which follows exactly the course of the adhesive plaster. These longitudinal strips are finally held in place by circular turns, some made a few inches above the ankle and some at the crista tibiæ. When this bandage has become dry and hard, the patient can assume any position without fear of disturbing his wound. The bandage must be removed if it becomes too loose or too tight, but in some cases the author had no occasion to meddle with it until the wound was firmly healed.

Hydated Tumor of the Kidney successfully treated by Aspiration.—Bradbury. (*British Med. Journal*, 1877, Oct. 6.) A boy, aged 8, was admitted into the hospital on July 5th, 1876. His only complaint was of an enlarged abdomen. A large tense elastic swelling occupied almost the whole of the left half of the abdomen, which was absolutely dull on percussion. Superiorly the percussion-dullness extended to within one inch and a half of the nipple in the nipple-line, and tracing it to the right, it became separated from the liver-dulness (right lobe) by a band of well-marked resonance. It then passed down, about one inch and a half to two inches to the right of the mesial line and lost itself below in the dullness of the (full) bladder. On tracing it to the left, the dullness reached as high as the left rib in the axillary line, but at this level it did not quite extend to the spine. The whole of the left hypochondrium was filled with the tumor, and there was complete dullness down to Poupart's ligament. The percussion was tympanitic over the rest of the abdomen. At the upper part of the tumor the "repercussion thrill" could be obtained. The heart's apex beat immediately beneath the nipple, just under the fourth rib; heart and lung sounds, urine and liver were normal.

On July 6th, the needle of an aspirator was introduced into the tumor, and forty-four ounces of hydatid fluid were drawn off. No hooklets were found in it. After the operation the boy vomited several times, had slight fever, and an eruption of urticaria; but no tenderness of the abdomen. The urine was found to contain albumen, due to the presence of pus. July 15th, the abdomen was enlarging again. When the boy was made to sit up in bed he complained of pain in the loins, and four of the lumbar spines were found to be prominent, and the skin over them reddened. They were very painful on pressure. The tumor was aspirated again, and thirty-one and a half ounces of a greenish opalescent fluid were withdrawn, which, in the latter stage of the operation, was flaky and apparently purulent. After standing, the fluid deposited two ounces of pus. Under the microscope, pus-cells and the heads of numerous echinococci armed with hooklets were detected. The boy vomited again several times after the operation, but no urticaria followed the second puncture. On July 25th and 26th small cysts with hooklets were found in the urinary sediment. From this time the patient became gradually better. In November he was discharged from the hospital and kept under observation for some months. When last seen, he was quite well, the abdomen being perfectly normal and the urine free from pus and albumen.

Epithelial Cancer Treated by Diluted Solution of Soda.—Prof. Busch.—(*Allgemeine Wiener Mediz. Zeitung*). The commencement of the epithelial cancer of the face is in many cases a simple proliferation of the epithelial cells of the external layers. Under certain circumstances the pressure of the external horny layer will cause atrophy of the papillæ underneath. In other cases, particularly in old age and in cases of constant irritation (chimney-sweeps), no epidermis is formed; the rete mucosum is exposed, and we have an *ulcus rodens*. If this horn-like layer of epidermal cells which obstructs the road for the succeeding cell formations is removed in a gentle manner, we can prevent the further growth of the cancers. This can be done by washing off the epidermoidal masses with a diluted solution of soda. Therefore, in the first stages of the facial and labial cancers, a cure can be accomplished by alkaline lotions.

This treatment is also sometimes successful in cancers that have existed for some time; and after the excision of a cancer a relapse can frequently be prevented by washing the scar with alkaline solutions.

Paget had observed that cancer may follow an excessive accumulation of the epithelial cells on the surface of the mamma.

Langenbeck also observed that he had seen very favorable results from the application of Emser thermal waters to cancerous ulcerations.

On the Regenerative Properties of the Periosteum and its Practical Application.—Von Langenbeck. (*Centralblatt fur Chirurgie* No. 22.)—When the periosteum of a bone has been removed, to a considerable extent it will be replaced by new periosteum, without necrosis of the bone occurring. If this new periosteum is deprived of its bone, the bone will also be replaced. This has been proven by experiments of Bernhard Heine.

Experience teaches Langenbeck that this re-produced periosteum possesses great vitality; and that for plastic operations the integument, that has become adherent to the bone, when removed with its periosteum, is of great value.

The author relates the history of two cases in which, through accident, the skulls were injured so that large openings were left in the cranial cavities. In these cases, L. used the integuments of the scars, with its periosteum, to cover and close the openings, and obtained the best results. He also related the case of a person who had lost a portion of the hard palate through syphilitic ulcerations. By using the tissue of the scars left, he succeeded in a partial restoration of the bone, while he had failed by using portions of the regular muco-periosteal lining or integument from unaffected portions of the mouth. Gangrene of these cicatricial flaps only occurred in anaemic individuals that should have had a previous tonic treatment.

